# Gambling harm prevention and harm reduction in online environments: a call for action

**DOI:** 10.1186/s12954-023-00828-4

**Published:** 2023-07-22

**Authors:** Virve Marionneau, Heidi Ruohio, Nina Karlsson

**Affiliations:** 1 Faculty of Social Sciences, Centre for Research on Addiction, Control, and Governance, University of Helsinki, Unioninkatu 33, 00014 Helsinki, Finland; 2 Finnish Institute for Health and Welfare, Mannerheimintie 166, 00271 Helsinki, Finland

**Keywords:** Gambling, Online, Ecosystem, Harm prevention, Harm reduction, Public health

## Abstract

**Background:**

Gambling is increasingly offered and consumed in online and mobile environments. The digitalisation of the gambling industry poses new challenges on harm prevention and harm reduction. The digital environment differs from traditional, land-based gambling environments. It increases many risk-factors in gambling, including availability, ease-of-access, but also game characteristics such as speed and intensity. Furthermore, data collected on those gambling in digital environments makes gambling offer increasingly personalised and targeted.

**Main results:**

This paper discusses how harm prevention and harm reduction efforts need to address gambling in online environments. We review existing literature on universal, selective, and indicated harm reduction and harm prevention efforts for online gambling and discuss ways forward. The discussion shows that there are several avenues forward for online gambling harm prevention and reduction at each of the universal, selective, and indicated levels. No measure is likely to be sufficient on its own and multi-modal as well as multi-level interventions are needed. Harm prevention and harm reduction measures online also differ from traditional land-based efforts. Online gambling providers utilise a variety of strategies to enable, market, and personalise their products using data and the wider online ecosystem.

**Conclusion:**

We argue that these same tools and channels should also be used for preventive work to better prevent and reduce the public health harms caused by online gambling.

## Background

The global online gambling market is growing rapidly. Market restrictions have been relaxed in many jurisdictions and the availability of gambling has increased in everyday lives. Recent legislative developments in the gambling field focus strongly on questions related to the regulation or re-regulation of online formats [1]. This digitalisation of gambling markets has also translated to an increasing interest in targeted harm prevention and reduction measures in online environments. Jurisdictions have focussed on questions including online advertisement and social responsibility, measures to block unlicensed online competition, as well as online-based treatment and information [1–6].

Effective harm reduction and harm prevention efforts are crucial to tackle gambling-related harms. This task is particularly pressing in online environments. A recent international meta-analysis showed that engagement in online gambling had the largest effect size for the risk of gambling-related problems [7]. Particularly young age and male gender are identified risk factors for gambling problems, particularly in online environments [7, 8]. Online casino games, fast online betting products, and emerging forms of online gambling such as skin betting, are causing an increasing share of gambling-related harms [9–12]. Online formats of gambling incorporate many harmful characteristics: high speed and intensity of play, bonuses and visible marketing, cross-selling of products, and a constant availability that has been further increased by the propagation of mobile gambling [cf. 13–15]. Even the same products provided online may be connected to higher levels of harms than if provided in land-based environments [16]. These harmful aspects are likely to be even further accentuated for mobile gambling (gambling taking place via mobile devices, usually using apps) which is more readily accessible than computer-based online gambling.

Much research has been conducted on the effectiveness of harm reduction and harm prevention efforts in land-based gambling, including several systematic reviews and a recent umbrella review [see 17–22]. These studies have shown that many harm prevention and harm reduction efforts can reduce consumption or harms in land-based environments, although effectiveness depends largely on implementation and how effects are measured. Impact studies have been scarce in gambling research and the effectiveness of many interventions has only been measured in terms of total consumption, or in some cases, changes in the number of individuals meeting psychometric criteria for problem gambling. Little research is available on the impacts of various harm reduction measures on the range of harms connected to gambling, such as suicidality, depression, or indebtedness.

Based on available research focussing mainly on land-based gambling, limiting availability and accessibility are among the most effective means to reduce total consumption. Availability-related measures include limiting the number of venues, venue hours, or access to cash or credit, as well as limiting accessibility with for example age limits [15, 17–20, 23]. Smoking bans and bans on the sale of alcohol in gambling venues have also led to reduced gambling consumption [15, 17, 18]. However, the high concentration of gambling spending may limit the impact of such measure on reducing harms and problematic gambling [15]. ‘Responsible gambling’ tools, such as precommitment and self-exclusion may also be effective to some extent. Their effectiveness is reduced by lacking identification of players in land-based environments [5, 17] and low utilisation rates in online environments [24] Pricing and taxation-related measures have received less empirical support [17, 23]. This is likely because individual gamblers are not always well-aware of the cost-of-play.

Whether insights from land-based gambling can be transferred to online gambling is not clear. There is a gap in our knowledge regarding what kind of harm prevention should be implemented in online environments and what kind of harm prevention is effective online. Gordon and Reith have argued that harm reduction efforts should be broadened to incorporate not only individual but also socio-cultural influences on gambling harms [14]. Such efforts should address structural and social factors such as spaces and places. The context of implementation is crucial to the effectiveness of measures [17]. This sensitivity towards contexts needs to be broadened to also incorporate the channels through which gambling as well as harm prevention and reduction efforts are implemented.

This paper addresses possibilities and challenges in implementing gambling harm prevention and harm reduction efforts in online environments. In the following, we chart universal, selective, and indicated harm reduction and harm prevention in online environments. As also argued by Simon et al. [25], a public health approach to harm reduction and harm prevention in gambling needs to target several levels to address the burden of problems for societies and individuals. We also discuss ways forward in improving harm reduction and harm prevention for gambling in online environments.

## Universal measures

Universal measures are generally targeted at the provision side. They include approaches that aim at reducing total consumption at a population-level. As has been shown in the field of alcohol and for some forms of gambling, reduced total consumption can translate to reduced harms [26, 27]. From a public health perspective, universal measures have been shown to be effective in terms of reducing and preventing population-level harms [15, 28].

*Availability* is a key issue in land-based gambling [15, 17]. Higher availability of gambling has been linked to higher prevalence of problem gambling [29], although other studies have also found support for the so-called adaptation hypothesis [30]. Effectiveness of availability reductions also depends on how extensive their implementation is [17]. Geographical availability of land-based gambling is relatively easy to implement. In online gambling, availability restrictions are not as straightforward, as particularly mobile gambling is characterised by 24 h availability. Examples of restricted availability in online environments nevertheless exist. For example, in Norway, the online casino operated by the national monopoly company Norsk Tipping, is closed between 3 and 7 am. In Finland, another monopoly country, deposits to the online casino website are not permitted between midnight and 6 am [31]. With regard to unregulated offshore gambling, availability has been restricted using a variety of blocking measures, such as IP/DNS blocking or payment blocking. These can be effective in preventing some traffic, but comprehensive implementation is resource-consuming [6]. For instance, gambling providers can easily change IP address or use fourth-party payment platforms (a kind of ‘mixing service’) to hide payment channels [32].

Much of online gambling takes place in mobile environments. App availability is therefore another question that needs novel solutions. The mobile gambling app ecosystem cannot be regulated merely by restricting gambling app availability in app stores because apps can also be downloaded directly from gambling websites or a variety of third-party app distribution sites. For example, an analysis of illegal gambling apps in China found over 6,000 websites offering gambling apps via 1,415 distribution sites [32]. We need more effort in targeting availability that is enabled via these complex ecosystems rather than focussing on gambling websites alone. Collaboration with app stores, developers, and distribution sites could offer a way forward.

*Exposure to marketing* is another universal issue in gambling harm reduction [17, 33]. Research evidence shows that there is a dose–response relationship between exposure to marketing and gambling behaviour [33]. Population studies have also associated marketing with problematic gambling [e.g. 34]. Marketing and adverting is often targeted at young individuals. A systematic review of the exposure of youth to gambling advertising [35] showed that particularly young men are affected by marketing. Marketing normalises gambling and reinforces harmful gambling behaviour. For young individuals, economic promotions constituted the most harmful type of marketing message [35].

In online environments, marketing takes increasingly complex forms. Limitations on the outlets, platforms, or even the contents of marketing messages are not enough in online environments. Online marketing is less universal and more targeted. For instance, social media heavily exposes people to gambling-related content.

Player data collected by gambling companies allows high levels of personalisation of marketing messages, including capitalising on sports fandom or cross-selling products [13, 36, 37]. Sponsorship deals with gambling companies also extend to e-sports and the world of online gaming [38]. Marketing on social media further increases exposure and can blur the line between marketing and other content [36]. Countries like France have already passed legislation to limit influencer marketing of harmful products in social media. It is crucial for regulations and harm prevention efforts to keep pace with the changing environment of online gambling marketing. Similarly, the data gambling companies collect on gamblers to propagate their marketing messages, could be put to better use in harm reduction, including using player data to target information on harmful play.

*Product characteristics* need to be better regulated to shift the responsibility in ‘responsible gambling’ from consumers to providers and regulators. Some countries, notably Sweden and the Netherlands, have included a ‘duty of care’ policy in their gambling legislation. However, the content of these policies currently focuses more on the monitoring of players rather than products [1]. Harmful product characteristics are not exclusive to online environments: Gambling products in land-based venues as well as digital channels are designed to be highly absorbing and addictive [4, 13, 39]. However, most current development in the market occurs online. For example, interactive features, gaming-like characteristics such as the possibility to ‘level up’, and online communities around gambling products increase the attractiveness (and potential harmfulness) of online gambling [13, 40]. Similarly, the online channel provides possibilities for increasingly fast-paced products, such as in-play betting [41]. There is a need to address these harmful characteristics more effectively with less harmful design [4]. To achieve this, some have called for new products to be vetted by regulators before their introduction [42]. B2B licensing may be one way of allowing regulators to license products in addition to operators [43].

*Information and education campaigns* have been identified as an insufficient solution to prevent gambling harms at a population level [15]. However, warning labels on products can be effective in reducing consumption if they are well designed. If the implementation of warning messages is poor, they may even encourage further consumption [44, 45]. Qualitative evidence from Australia [e.g. 46] suggests that warnings highlighting individual responsibility, such as ‘gamble responsibly’ are not considered effective. Rather, they are seen to absolve the gambling industry of its own responsibility. Instead of individual responsibility, effective warning messages should highlight the severe harms that gambling causes, similarly to warning labels on tobacco products [46]. Targeting information campaigns to particular social groups may also be more effective. For example, in New Zealand, recent campaigns have been targeted at the Māori community [47].

Targeted or dynamic forms of information are easy to implement online also for other groups. Data could allow personalised warnings and other information campaigns in online environments, in the same way as marketing campaigns are targeted and personalised. This can be done at a consumer-level but also at a more universal level of customer segments. Literature on online health promotion and health awareness suggests that social media and ‘influencer’ created content may be effective particularly among young people [48, 49]. For example, an ongoing European Union (EU) funded project Gambling Free Feed aims at influencing young people’s attitudes towards gambling and developing a model for harm prevention [50].

## Selective measures

Selective measures are aimed at vulnerable populations with an increased risk of developing gambling-related problems. The definition of vulnerable populations can be flexible. One review study identified at least seven vulnerable groups to gambling harms: young people, older adults, women, veterans, indigenous peoples, prisoners, and low-income groups [51]. In online environments, selective measures can also be targeted based on gambling-behaviour rather than demographic or socio-economic characteristics. Behaviour-based selective measures require identification. As identification is widely available in online environments, these forms of prevention are easier to implement than in land-based gambling.

*Age restrictions* are among the most universally applied gambling policies to prevent harms [e.g. 1, 52]. For age restrictions to be effective, age verification needs to be systematically implemented using for example electronic identification [44, 53]. This is easy to implement online. Cross-checking the identity of consumers from national identity databases and personal identification systems is a particularly good practice to prevent access for underaged (or self-excluded) players [44, 54]. In addition to preventing underage gambling, identification in online environments can be used to target more stringent play limitations on emerging adults. For example, the Swedish gambling provider, Svenska Spel, has a lower deposit limit for under 20-year-olds than for older players [55].

*Pre-commitment* includes tools aimed at gamblers to control their own gambling, including limit-setting and self-exclusion. These can be useful in restricting and preventing problematic gambling [5, 22]. The effectiveness of mandatory pre-commitment has received more empirical support while evidence on the effectiveness of voluntary pre-commitment systems has been inconsistent [17, 20, 21]. Mandatory identification in online environments also allows developing and implementing global limit setting and centralised, cross-provider self-exclusion registries.

Self-exclusion has been researched in various contexts, but results have depended on implementation [22]. It is likely that website-specific self-exclusions are less effective than centralised systems. For example, Sweden, the Netherlands, and Germany have recently introduced national self-exclusion registries. Research shows that cross-provider self-exclusion registries have a wide reach, but the existence of unlicensed gambling provision in online environments reduces their effectiveness [56]. In addition to self-exclusions, online operators can provide, or be legally obliged to provide temporary exclusions, such as panic buttons [e.g. 57]. Those who want to restrict their own online gambling can also download blocking software designed to prevent access to online gambling sites on computers or mobiles. These types of software have been found to block up to 99% of gambling sites, including offshore provision [58].

Similarly to self-exclusions, the effectiveness of limit-setting depends on implementation. Limit-setting can be mandatory or voluntary, and consist of different forms (play limit, deposit limit, bet limit, loss limits; [cf. 59]). Overall, limits are well accepted by players. A survey study of Swedish active online gamblers (*N* = 10,200) showed that 95.6% of all gamblers viewed limit-setting positively. The same study showed that those who were not experiencing problems with their gambling, were also not inconvenienced by pre-commitment measures [60]. However, there was no statistically significant relationship between limit-setting and the proportion of players with a positive net loss [60]. Mandatory limits are, in general, more effective. Most players also stop when reaching their limits [5]. Limits must be reasonable to be effective. Without legislatively mandated maximum limits, providers can allow limits up to millions of euros [61]. In online environments, it has become possible to establish reasonable, cross-provider limit-setting. For example, Germany upholds a centralised system across all licensed gambling providers that maintains a cross-provider deposit limit of 1000 euros a month.

In addition to providers, online payment gateway services can also be employed to track gambling accounts across gambling platforms, possibly including those that are not licensed. Gambling websites and apps use third-party and even fourth-party payment services for improved privacy [32]. These payment solution services could also be used to track possibly harmful purchase patterns or to control cross-provider limit-setting [62].

*Pop-ups and targeted information tools* have been studied particularly in land-based EGM (electronic gambling machine) gambling, but the findings also apply to online environments. Pop-ups can be utilised, for example, to remind gamblers about pre-set monetary or time limits [22, 42, 63]. Pop-ups can be effective in limiting gambling consumption, but effectiveness depends on implementation [21, 22]. Overall, more engaging and personalised pop-ups are connected to higher adherence to limits [22]. It would be necessary to conduct more empirical research on the effects of pop-ups and targeted information in online environments and among different populations [cf. 64, 65. For example, younger players may be more receptive to pictures.

Besides pop-ups, other nudges can also be used to help gamblers make better decisions. In behavioural studies, nudges are understood as small changes in the environment that encourage healthier or safer choices [66]. For instance, less harmful gambling products could have a more prominent place on a gambling website. However, as argued by Newall et al., gambling companies also use the same strategies for ‘sludges’ that encourage harmful behaviour [67]. For example, withdrawals from gambling accounts can be made more difficult than deposits, discouraging players from accessing their winnings [66]. Gambling apps also use a variety of third-party services, such as push notification services that are not currently regulated but that could be targeted for improved regulation and harm prevention [32].

Informative tools can also prime analytical thinking [22]. This can be done for example by showing informative videos on odds or to correct possibly erroneous cognitions of players. Some research is available, although these have shown little effect [67]. For example, Wohl et al. [68] showed educational animations in real-life gambling settings but did not find any statistically significant difference between the group that watched the video and the group that did not. These kinds of tools could be easily implemented in online environments, although they are unlikely to be effective without other accompanying measures.

## Indicated interventions

Indicated harm reduction targets individuals with a particular risk of developing gambling-related problems. The online environment offers new opportunities to identify and target indicated interventions as well as treatment to those who have already been affected by gambling harms.

*Identification of problematic play* is possible in online environments using algorithmic solutions that recognise play or spending patterns. Identified online gambling allows the use of machine learning and artificial intelligence (AI) for effective identification of harmful behaviours. A growing body of research employs AI to predict for example self-exclusions, self-reported problem gambling, or limit setting in players [69–72]. For example, Ukhov et al. [73] used AI and found that the number of daily bets and gambling on mobile devices predicted problematic gambling among online sports bettors, while the duration of gambling sessions, amounts deposited, and the use of desktop computers predicted problematic gambling among online casino players. Gambling operators are also developing their own tools to identify and intervene in harmful gambling patterns, often as part of their duty of care obligations [cf. 1, 74. However, regulators and independent researchers need to be involved in these AI-based developments. Otherwise, behavioural tools risk being used as so-called dark nudges or sludges [75] that aim at manipulating players to act in ways that are not in their best interest [76].

*Feedback interventions* refer to personalised actions that are taken based on identified problematic gambling patterns. Studies on feedback interventions have found that personalised feedback can lead to reduced gambling [20]. One way of accomplishing this is by contacting players who have been identified as likely to have problematic gambling patterns. A study conducted in Sweden found that motivational interview interventions by telephone, combined with personalised feedback led to reduced theoretical loss (i.e. the amount of money a player is statistically expected to lose) among the target group [77]. Another Swedish study showed that telephone interventions were more effective than letters [78]. The contents of feedback interventions are likely to impact results, and those with more severe gambling-related problems may respond better to these interventions [79].

In addition to telephone calls, the online environment also allows many technological interventions, ranging from simple personalised pop-ups to more important changes in the gambling interface. For example, the gambling platform interface can be changed based on consumption patterns to incorporate personalised information on harms and to hide the products that are causing these harms. This, too, would need to be a centralised and cross-provider practice.

*Therapeutic interventions* in online environments include approaches such as web-based psychotherapy or counselling, and online support forums [80]. The characteristics of treatment interventions in online environments have already received some research attention [22, 80], with most research focussing on computer-assisted or online therapy programmes for disordered gambling. Overall, studies have shown mixed results. For example, Luquiens et al. [81] investigated the effects of an email-based self-help programme, grounded in cognitive behavioural therapy. They found no significant difference regarding observable behaviour, gambling problems, or financial harm. Carlbring et al. [82] studied a computer-assisted therapy programme, combined with telephone support. The intervention resulted in favourable changes in pathological gambling behaviour, anxiety, depression, and quality of life. Another study by Neighbors et al. [83] similarly combined computer-delivered intervention and personalised feedback. The study showed positive effects on gambling behaviour.

During the Covid-19 pandemic, many help and support services were moved to online-only environments. While little research is available on the effects and effectiveness of these changes in delivery, qualitative accounts from service users and providers imply that implementing online-based therapeutic interventions may be challenging, particularly in terms of client engagement and privacy concerns [84]. However, some have also considered online-based treatment positively, as a more accessible, low-threshold option [85]. Similar findings have been reported in the field of mental health where face-to-face and/or web-based support has been an important feature of online interventions in regards of programme outcomes and participant completion [see 86].

## Conclusions

This paper has provided an overview of possible avenues and challenges for harm prevention and harm reduction for gambling in online environments. We have charted universal, selective, and indicated measures based on existing research and examples. We have argued that each of these levels offer several possibilities for online gambling harm prevention and reduction that have not yet been sufficiently utilised. The overview has also shown that harm prevention and reduction in online environments differs from that in land-based environments. This observation supports the vital importance of the gambling contexts, including the channel of gambling, in designing and implementing effective measures to reduce the negative public health impacts of gambling [cf. [14].

In comparison with land-based environments, the online environment incorporates several challenges, including wider availability, targeted and data-driven marketing and offer, and a complex ecosystem of provision that makes regulation difficult and easy to escape: Information and communication tools are widely employed by gambling operators to enable, market, and personalise the gambling experience for consumers [13, 36, 87]. Many of these same characteristics could and should be used in preventive work. For example, data can also be used to target harm prevention measures while payment channels can provide possible solutions for more centralised efforts to track consumption and implement limits. The wider online ecosystem can also expand our definition of duty of care to include not only gambling providers, but also other actors in the production chain and surrounding ecosystem.

A harm reduction approach differs from the ‘responsible gambling’ approach in that it does not primarily focus on player responsibility, the preservation of interests of recreational players and the industry [25], but on the fuller range of public health implications. However, no measure is sufficient on its own. Focus needs to be on multi-modal interventions, as systematic interventions and policies are usually more effective than stand-alone interventions [19]. A solid body of research shows that population-level, universal, actions are the most effective in gambling as well as in other sectors [e.g. 15, 80, 88, 6,89. Yet, harm prevention and reduction action need to also include selective and indicated measures to help those who have already been or are at risk of being afflicted by gambling harms. Online environments provide improved opportunities to recognise those at risk, and online interventions can offer a low-threshold option to access treatment, particularly if combined with personal support by, for example, telephone.

More research is still needed to establish good practices for harm prevention and reduction for online gambling. Effective harm prevention and reduction in online environments does not concern the gambling research field only. Online sales of other potentially harmful products, such as alcohol or tobacco, may share similar challenges from a harm prevention point of view. In addition, researchers and harm prevention professionals need to collaborate more extensively with IT specialists to establish how the online environment works and how it can be utilised [cf. 6]. We also need to study the role of emerging marketing channels, including targeted advertisement, push notifications, social media presence, or the harmful effects of ‘sludges’ [90]. These issues need better regulation, but at the same time, the possibilities of the same targeted strategies need to be more extensively explored as tools for harm prevention and reduction.

The global increase in digital services, including online gambling, is a cross-sectoral and transnational issue that should be addressed by international bodies such as the European Union, but also target commercial actors, including payment services, software developers, and the server infrastructure. Effective measures for online content filtering and blocking require coherent legal actual across jurisdictions and fields [cf. 91]. In line with the health in all policies approach, gambling harms need to be targeted as a public health issue on all levels, to reduce and prevent the burden of harms that gambling causes on individuals and societies.

## Data Availability

Data sharing is not applicable to this article as no datasets were generated or analysed during the current study.

## Abbreviations

AI: Artificial intelligence
DNS: Domain name system
EGM: Electronic gambling machine
EU: European Union
IP: Internet protocol

## References

[1] Ukhova D, Marionneau V, Nikkinen J, Wardle H. Embracing public health approaches to gambling? A review of global legislative and regulatory trends (forthcoming). 10.1016/S2468-2667(23)00221-9 PMC10927617 37944544

[2] Hörnle J, Schmidt-Kessen M, Littler A, Padumadasa E (2019). Regulating online advertising for gambling-once the genie is out of the bottle…. Inform Commun Technol Law.

[3] Hörnle J, Littler A, Schmidt-Kessen M, Tyson G, Padumadasa E (2019). Evaluation of regulatory tools for enforcing online gambling rules and channelling demand towards controlled offers. Publ Office.

[4] Newall PW (2023). Reduce the speed and ease of online gambling in order to prevent harm. Addiction.

[5] Delfabbro PH, King DL (2021). The value of voluntary vs. mandatory responsible gambling limit-setting systems: a review of the evidence. Int Gambl Stud.

[6] Egerer M, Marionneau V (2023). Blocking measures against offshore online gambling: a scoping review. Int Gambl Stud.

[7] Allami Y, Hodgins DC, Young M, Brunelle N, Currie S, Dufour M, Nadeau L (2021). A meta-analysis of problem gambling risk factors in the general adult population. Addiction.

[8] Mora-Salgueiro J, García-Estela A, Hogg B, Angarita-Osorio N, Amann BL, Carlbring P, Colom F (2021). The prevalence and clinical and sociodemographic factors of problem online gambling: a systematic review. J Gambl Stud.

[9] Booth L, Anderson AS, White V, Pierce H, Moodie R, Pettigrew S (2021). Public perceptions of harm for nine popular gambling products. J Gambl Stud.

[10] Lind K, Marionneau V, Järvinen-Tassopoulos J, Salonen AH (2021). Socio-demographics, gambling participation, gambling settings, and addictive behaviors associated with gambling modes: A population-based study. J Gambl Stud.

[11] Raybould JN, Larkin M, Tunney RJ (2021). Is there a health inequality in gambling related harms? A systematic review. BMC Public Health.

[12] Wardle H, Tipping S (2023). The relationship between problematic gambling severity and engagement with gambling products: longitudinal analysis of the Emerging Adults Gambling Survey. Addiction.

[13] Wardle H (2021). Games without frontiers? Socio-historical perspectives at the gaming/gambling intersection. Springer Nature.

[14] Gordon R, Reith G (2019). Gambling as social practice: a complementary approach for reducing harm?. Harm Reduct J.

[15] Sulkunen P, Babor T, Cisneros Örnberg J, Egerer M, Hellman M, Livingstone C, Marionneau V, Nikkinen J, Orford J, Room R, Rossow I (2019). Setting Limits: Gambling, Science, and Public Policy.

[16] Gainsbury SM, Angus DJ, Blaszczynski A (2019). Isolating the impact of specific gambling activities and modes on problem gambling and psychological distress in internet gamblers. BMC Public Health.

[17] Velasco V, Scattola P, Gavazzeni L, Marchesi L, Nita IE, Giudici G (2021). Prevention and harm reduction interventions for adult gambling at the local level: an umbrella review of empirical evidence. Int J Environ Public Health.

[18] Tanner J, Drawson AS, Mushquash CJ, Mushquash AR, Mazmanian D (2017). Harm reduction in gambling: a systematic review of industry strategies. Addict Res Theory.

[19] Livingstone C, Rintoul A, de Lacy-Vawdon C, Borland R, Dietze P, Jenkinson R. Identifying effective policy interventions to prevent gambling-related harm. Melbourne VIC Australia: Victorian Responsible Gambling Foundation; 2019. https://responsiblegambling.vic.gov.au/documents/640/Livingstone-identifying-effective-policy-interventions-June-2019.pdf

[20] McMahon N, Thomson K, Kaner E, Bambra C (2019). Effects of prevention and harm reduction interventions on gambling behaviours and gambling related harm: an umbrella review. Addict Behav.

[21] Forsström D, Spångberg J, Petterson A, Brolund A, Odeberg J (2021). A systematic review of educational programs and consumer protection measures for gambling: an extension of previous reviews. Addict Res Theory.

[22] Fiskaali A, Westh Stenbro A, Marcussen T, Tröllund RM (2022). Preventive interventions and harm reduction in online and electronic gambling: a systematic review. J Gambl Stud.

[23] Gainsbury SM, Blankers M, Wilkinson C, Schelleman-Offermans K, Cousijn J (2014). Recommendations for international gambling harm-minimisation guidelines: comparison with effective public health policy. J Gambl Stud.

[24] Procter L, Angus DJ, Blaszczynski A, Gainsbury SM (2019). Understanding use of consumer protection tools among Internet gambling customers: utility of the theory of planned behavior and theory of reasoned action. Addict Behav.

[25] Simon O, Savary JF, Guarrasi G, Dickson C. Defining harm reduction as part of a public health approach towards gambling. In Harm Reduction for Gambling. 2019:71–77. Routledge. 10.4324/9780429490750

[26] Rossow I (2019). The total consumption model applied to gambling: empirical validity and implications for gambling policy. Nordic Stud Alcohol Drugs.

[27] Rossow I, Mäkelä P (2021). Public health thinking around alcohol-related harm: Why does per capita consumption matter?. J Stud Alcohol Drugs.

[28] van Schalkwyk MC, Petticrew M, Cassidy R, Adams P, McKee M, Reynolds J, Orford JA (2021). public health approach to gambling regulation: countering powerful influences. Lancet Public Health.

[29] Storer J, Abbott M, Stubbs J (2009). Access or adaptation? A meta-analysis of surveys of problem gambling prevalence in Australia and New Zealand with respect to concentration of electronic gaming machines. Int Gambl Stud.

[30] Abbott M (2017). Gambling and gambling harm in New Zealand: a 28-year case study. Int J Ment Health Addict.

[31] Heiskanen M, Hellman M, Jaakkola T, Kinnunen, J, Levitski A, Lerkkanen T, Marionneau V, Oksanen A, Pajula M & Salonen A. Rahapeliautomaattien ja automaattipelien haittojen hallinta: Rahapelien toimeenpanosta aiheutuvien haittariskien ja haittojen arviointiryhmän päätelmät haittojen hallinnan tehostamistarpeista ja ryhmänohjauksessa tehty selvitys. Helsinki: Sosiaali- ja terveysministeriö; 2020. http://hdl.handle.net/10138/321365

[32] Gao Y, Wang H, Li L, Luo X, Xu G, Liu X. Demystifying illegal mobile gambling apps. In Najork M, Tang J, Zia L, editors, Proceedings of The World Wide Web Conference WWW 2021. New York NY USA: Association for Computing Machinery (ACM). 2021. p. 1447–1458. doi: 10.1145/3442381.3449932

[33] McGrane E, Wardle H, Clowes M, Blank L, Pryce R, Field M, Goyder E (2023). What is the evidence that advertising policies could have an impact on gambling-related harms? A systematic umbrella review of the literature. Public Health.

[34] Syvertsen A, Erevik EK, Hanss D, Mentzoni RA, Pallesen S (2022). Relationships between exposure to different gambling advertising types, advertising impact and problem gambling. J Gambl Stud.

[35] Labrador FJ, Estupiñá FJ, Vallejo-Achón M, Sánchez-Iglesias I, González-Álvarez M, Fernández-Arias I, Bernaldo-de-Quirós M (2021). Exposure of adolescents and youth to Gambling advertising: a systematic review. An Psicol..

[36] Guillou-Landreat M, Gallopel-Morvan K, Lever D, Le Goff D, Le Reste JY (2021). Gambling marketing strategies and the internet: What do we know? A systematic review. Front Psychiatry..

[37] Newall PW, Moodie C, Reith G, Stead M, Critchlow N, Morgan A, Dobbie F (2019). Gambling marketing from 2014 to 2018: a literature review. Curr Addict Rep.

[38] Biggar B, Zendle D, Wardle H (2023). Targeting the next generation of gamblers? Gambling sponsorship of esports teams. J Public Health.

[39] Schüll ND (2012). Addiction by design.

[40] Sirola A, Kaakinen M, Oksanen A (2018). Excessive gambling and online gambling communities. J Gambl Stud.

[41] Lopez-Gonzalez H, Griffiths MD, Estévez A (2020). In-play betting, sport broadcasts, and gambling severity: a survey study of Spanish sports bettors on the risks of betting on sport while watching it. Commun Sport.

[42] van Schalkwyk MC, Cassidy R, Petticrew M, McKee M (2023). Harm built in—why the gambling industry needs a Silent Spring moment. BMJ.

[43] Riksdagen (2022). Regeringens proposition 2021/22:242. https://data.riksdagen.se/fil/F1F46CBC-913C-4358-98E4-9DE535BEA0B7

[44] Shi J, Colder Carras M, Potenza MN, Turner NE (2021). A perspective on age restrictions and other harm reduction approaches targeting youth online gambling, considering convergences of gambling and videogaming. Front Psychiatry..

[45] Mouneyrac A. Messages de prévention promouvant le Jeu responsable: une injonction paradoxale dans les jeux de hasard et d'argent (Doctoral dissertation, Université Toulouse le Mirail-Toulouse II). 2019.

[46] Pitt H, Thomas SL, Randle M, Cowlishaw S, Arnot G, Kairouz S, Daube M (2022). Young people in Australia discuss strategies for preventing the normalisation of gambling and reducing gambling harm. BMC Public Health.

[47] Health New Zealand. Health Promotion. Reflecting on culture to protect communities from harm. 2022. https://www.hpa.org.nz/campaign/nans-song

[48] Latha K, Meena KS, Pravitha MR, Dasgupta M, Chaturvedi SK (2020). Effective use of social media platforms for promotion of mental health awareness. J Edu Health Promot.

[49] Korda H, Itani Z (2013). Harnessing social media for health promotion and behavior change. Health Promot Pract.

[50] Sosped foundation. Gambling Free Feed -project. https://sosped.fi/kansainvalinen-yhteistyo/gff/. Accessed 20 April 2023.

[51] Sharman S, Butler K, Roberts A (2019). Psychosocial risk factors in disordered gambling: A descriptive systematic overview of vulnerable populations. Addict Behav.

[52] Sichali JM, Bunn C, McGee D, Marionneau VK, Yendork JS, Glozah F, Reith G (2023). Regulation of gambling in Sub-Saharan Africa: findings from a comparative policy analysis. Public Health.

[53] Andrade M, Sharman S, Xiao LY, Newall PW (2022). Safer gambling and consumer protection failings among 40 frequently visited cryptocurrency-based online gambling operators. Psychol Addict Behav.

[54] Nash V, O'Connell R, Zevenbergen B, Mishkin A. Effective age verification techniques: Lessons to be learnt from the online gambling industry. 2012. Available at SSRN 2658038.

[55] Svenska Spel. (2023). https://spela.svenskaspel.se/spelkoll/andring-av-manadsgranser.

[56] Håkansson A, Åkesson G (2022). Multi-operator Self-exclusion as a Harm Reduction Measure in Problem Gambling: Retrospective Clinical Study on Gambling Relapse Despite Self-exclusion. JMIR Mental Health..

[57] Forsström D, Cisneros ÖJ (2019). Responsible gambling in practice: A case study of views and practices of Swedish oriented gambling companies. Nordic Stud Alcohol Drugs.

[58] Talbot A. Evaluating online blocking software. Report prepared for GambleAware (2018). https://www.begambleaware.org/sites/default/files/2020-12/blocking-software-evaluation-findings-report%20%281%29.pdf

[59] Auer M, Hopfgartner N, Griffiths MD (2018). The effect of loss-limit reminders on gambling behavior: a real-world study of Norwegian gamblers. J Behav Addict.

[60] Ivanova E, Magnusson K, Carlbring P (2019). Deposit Limit Prompt in Online Gambling for Reducing Gambling Intensity: A Randomized Controlled Trial. Front Psychol.

[61] Marionneau V, Järvinen-Tassopoulos J (2017). Consumer protection in licensed online gambling markets in France: the role of responsible gambling tools. Addict Res Theory.

[62] Lakew N (2022). “Show Me the Money”: preliminary lessons from an implementation of intervention tools at the payment gateway level. J Gambl Stud.

[63] Wohl MJA, Parush A, Kim HS, Warren K (2014). Building it better: applying human–computer interaction and persuasive system design principles to a monetary limit tool improves responsible gam­bling. Comput Hum Behav.

[64] Ginley MK, Whelan JP, Keating HA, Meyers AW (2016). Gambling warning messages: the impact of winning and losing on message reception across a gambling session. Psychol Addict Behav.

[65] Harris A, Griffiths MD (2017). A critical review of the harm-minimisation tools available for elec­tronic gambling. J Gambl Stud.

[66] Newall PW, Rockloff MJ (2022). Promoting safer gambling via the removal of harmful sludge: a view on how behavioural science's ‘nudge’concept relates to online gambling. Addiction.

[67] Armstrong T, Rockloff M, Browne M, Blaszczynski A (2020). Encouraging gamblers to think critically using generalised analytical priming is ineffective at reducing gambling biases. J Gambl Stud.

[68] Wohl MJA, Gainsbury S, Stewart MJ, Sztainert T (2013). Facilitating responsible gambling: the relative effectiveness of education-based animation and monetary limit setting pop-up messages among electronic gaming machine players [article]. J Gambl Stud.

[69] Auer M, Griffiths MD (2022). Using artificial intelligence algorithms to predict self-reported problem gambling with account-based player data in an online casino setting. J Gambl Stud.

[70] Auer M, Griffiths MD (2022). Predicting limit-setting behavior of gamblers using machine learning algorithms: a real-world study of Norwegian gamblers using account data. Int J Ment Health Addict.

[71] Louderback ER, LaPlante DA, Currie SR, Nelson SE (2021). Developing and validating lower risk online gambling thresholds with actual bettor data from a major internet gambling operator. Psychol Addict Behav.

[72] Finkenwirth S, MacDonald K, Deng X, Lesch T, Clark L (2021). Using machine learning to predict self-exclusion status in online gamblers on the PlayNow.com platform in British Columbia. Int Gambl Stud.

[73] Ukhov I, Bjurgert J, Auer M, Griffiths MD (2021). Online problem gambling: a comparison of casino players and sports bettors via predictive modeling using behavioral tracking data. J Gambl Stud.

[74] McAuliffe WH, Louderback ER, Edson TC, LaPlante DA, Nelson SE (2022). Using “markers of harm” to track risky gambling in two cohorts of online sports bettors. J Gambl Stud.

[75] Newall PW (2019). Dark nudges in gambling. Addict Res Theory.

[76] Jonsson J. Manipulativ speldesign: En jämförelse av dark patterns förekomst på olika spelplattformar. 2022.

[77] Jonsson J, Hodgins DC, Munck I, Carlbring P (2019). Reaching out to big losers: a randomized controlled trial of brief motivational contact providing gambling expenditure feedback. Psychol Addict Behav.

[78] Jonsson J, Hodgins DC, Munck I, Carlbring P (2020). Reaching out to big losers leads to sustained reductions in gambling over 1 year: a randomized controlled trial of brief motivational contact. Addiction.

[79] Cunningham JA, Hodgins DC, Toneatto T, Murphy M (2012). A randomized controlled trial of personalized feedback intervention for problem gamblers. PLoS ONE.

[80] Paterson M, Whitty M, Boyer C (2021). An overview of digital and online strategies to reduce gambling harm. Health Promot J Austr.

[81] Luquiens A, Tanguy ML, Lagadec M, Benyamina A, Aubin HJ, Reynaud M (2016). The efficacy of three modalities of internet-based psychotherapy for non-treatment-seeking online problem gamblers: a randomized controlled trial. J Med Internet Res.

[82] Carlbring P, Smit F (2008). Randomized trial of internet-delivered self-help with telephone support for pathological gamblers. J Consult Clin Psychol.

[83] Neighbors C, Rodriguez LM, Rinker DV, Gonzales RG, Agana M, Tackett JL, Foster DW (2015). Efficacy of personalized normative feedback as a brief intervention for college student gambling: a randomized controlled trial. J Consult Clin Psychol.

[84] Turner E, Shi J, Agic B, van der Maas M, Agasee S & Watson T. The adaptation to COVID-19 by problem gambling and mental health treatment providers in Canada: a brief report. J Gambling Issues, online first (2023).

[85] Marionneau V, Järvinen-Tassopoulos J (2022). Treatment and help services for gambling during COVID-19: experiences of gamblers and their concerned significant others. Nordic Stud Alcohol Drugs.

[86] Clarke AM, Kuosmanen T, Barry MM (2015). A systematic review of online youth mental health promotion and prevention interventions. J Youth Adolescence.

[87] Thomas S, van Schalkwyk MC, Daube M, Pitt H, McGee D & McKee M. Protecting children and young people from contemporary marketing for gambling. Health Promotion International. 2023;38(2):daac194. 10.1093/heapro/daac194 10.1093/heapro/daac194 PMC10024482 36932993

[88] Price A, Hilbrecht M, Billi R (2021). Charting a path towards a public health approach for gambling harm prevention. J Public Health.

[89] Folkhälsomyndigheten (2019). Spelproblem går att förebygga. https://www.folkhalsomyndigheten.se/publikationer-och-material/publikationsarkiv/s/spelproblem-gar-att-forebygga/

[90] Newall PW, Walasek L, Ludvig E & Rockloff M. Nudge versus sludge in gambling warning labels: How the effectiveness of a consumer protection measure can be undermined. 10.31234/osf.io/gks2h

[91] Rojszczak M (2022). Online content filtering in EU law-A coherent framework or jigsaw puzzle?. Comput Law Secur Rev.

